# Supplementary material for: Trichoderma versus Fusarium—Inhibition of Pathogen Growth and Mycotoxin Biosynthesis
Source: Molecules. 2022 Nov 23;27(23):8146. doi: 10.3390/molecules27238146 (PMC9735881; doi:10.3390/molecules27238146)
Supplement: Supplementary file 1 [file molecules-27-08146-s001.zip › molecules-1991069-supplementary.pdf]

Supplementary Materials: *Trichoderma* versus *Fusarium* – inhibition of pathogen growth and mycotoxins synthesis

Marta Modrzewska, Lidia Błaszczyk, Łukasz Stępień, Monika Urbaniak, Agnieszka Waśkiewicz, Tomoya Yoshinari and Marcin Bryła

Table S1. Ions mass and retention time for individual mycotoxins

Mycotoxin	Molecular ion (m/z)	Retention time (min)
NIV	313.127 $[M+H]^+$	1.70
NIV-3G	492.207 $[M+NH_4]^+$	1.80
FUS-X	355.138 $[M+H]^+$	5.04
DON	297.132 $[M+H]^+$	3.21
DON-3G	503.178 $[M+COO]^-$	3.89
3-AcDON	339.841 $[M+H]^+$	5.80
15-AcDON	339.841 $[M+H]^+$	5.80
ZEN	317.139 $[M-H]^-$	9.44
α-ZOL	319.155 $[M-H]^-$	9.02
β-ZOL	319.155 $[M-H]^-$	8.31
ZEN-14G	479.193 $[M-H]^-$	7.58
ZEN-14S	397.097 $[M-H]^-$	7.24

Table S2. Correlation coefficient, analytical ranges, limit of detection (LOD), and limit of quantification (LOQ)

Analyte	Correlation Coefficient (R^2)	Analytical ranges (mg/kg)	LOQ (mg/kg)	LOD (mg/kg)
NIV	0.9949	0.10-8.00	0.15	0.05
NIV-3G	0.9977	0.10-3.20	0.16	0.05
FUS-X	0.9972	0.10-3.20	0.12	0.04
DON	0.9981	0.10-8.00	0.12	0.04
DON-3G	0.9966	0.10-3.20	0.18	0.06
3- + 15- AcDON	0.9967	0.03-1.60	0.03	0.01
ZEN	0.9987	0.10-8.00	0.03	0.01
α-ZOL	0.9972	0.03-0.80	0.03	0.01
β-ZOL	0.9949	0.01-0.40	0.03	0.01
ZEN-14G	0.9921	0.10-4.00	0.18	0.06
ZEN-14S	0.9920	0.10-4.00	0.24	0.08

Table S3. Recovery rate (R%) and method repeatability (expressed as relative standard deviation, RSD%) for individual analytes at four different fortification levels.

Analyte	Fortification level (mg/kg) (n = 5)	R (%)	RSD (%)
NIV	1	95	13.6
	2	75	17.2
	4	88	10.0
	8	86	10.0
NIV-3G	0.4	98	11.7
	0.8	88	9.7
	1.6	94	4.2
	3.2	79	7.1
FUS-X	0.4	72	12.5
	0.8	77	14.0
	1.6	78	4.0
	3.2	85	5.0
DON	1	100	9.4
	2	93	11.6
	4	104	8.3
	8	81	11.0
DON-3G	0.4	84	14.5
	0.8	82	9.1
	1.6	90	2.0
	3.2	92	8.0
3- + 15- AcDON	0.2	122	3.1
	0.4	109	4.9
	0.8	107	6.4
	1.6	102	10.3
ZEN	1	84	10.2
	2	79	9.1
	4	86	4.0
	8	94	4.1
α-ZOL	0.1	84	22.0
	0.2	77	12.2
	0.4	90	4.6
	0.8	95	4.7
β-ZOL	0.05	93	14.1
	0.1	80	16.9
	0.2	71	2.3
	0.4	76	6.0
ZEN-14G	0.5	70	34.8
	1	81	8.3
	2	86	4.3
	4	79	3.6
ZEN-14S	0.5	77	13.2
	1	110	21.7
	2	111	16.9
	4	112	6.2
